# Evaluating Muscle Activation Models for Elbow Motion Estimation

**DOI:** 10.3390/s18041004

**Published:** 2018-03-28

**Authors:** Tyler Desplenter, Ana Luisa Trejos

**Affiliations:** 1 Department of Electrical and Computer Engineering, Western University, London, ON N6A 5B9, Canada; tdesplen@uwo.ca; 2 Canadian Surgical Technologies and Advanced Robotics, Lawson Health Research Institute, London, ON N6A 5A5, Canada

**Keywords:** electromyography, elbow model, muscle activation model, estimation accuracy, computational resources

## Abstract

Adoption of wearable assistive technologies relies heavily on improvement of existing control system models. Knowing which models to use and how to improve them is difficult to determine due to the number of proposed solutions with relatively little broad comparisons. One type of these models, muscle activation models, describes the nonlinear relationship between neural inputs and mechanical activation of the muscle. Many muscle activation models can be found in the literature, but no comparison is available to guide the community on limitations and improvements. In this research, an EMG-driven elbow motion model is developed for the purpose of evaluating muscle activation models. Seven muscle activation models are used in an optimization procedure to determine which model has the best performance. Root mean square errors in muscle torque estimation range from 1.67–2.19 Nm on average over varying input trajectories. The computational resource demand was also measured during the optimization procedure, as it is an important aspect for determining if a model is feasible for use in a particular wearable assistive device. This study provides insight into the ability of these models to estimate elbow motion and the trade-off between estimation accuracy and computational demand.

## 1. Introduction

In a world where the use of wearable assistive devices is becoming increasingly plausible, there is a need for the continued advancement of the systems responsible for controlling these devices. One avenue to be explored in this evolutionary chain is regarding the modelling of relationships between biological signals, such as electromyography (EMG), and joint motion. The benefit of improving these models is the enhancement in control of wearable mechatronic devices, such as those used in rehabilitation of musculoskeletal elbow injuries. Being able to know which motion the user desires to complete will enable the accurate control of the device’s behaviour. Models describing the relationships between desired and produced motion, detected through EMG and motion sensors, respectively, have been proposed for wearable systems, such as the NEUROExos elbow exoskeleton or the elbow arm support developed by Lobo-Prat et al. [1,2]. However, the limited comparison of model performance makes it difficult to determine if a model is appropriate for a particular wearable device or application.

**Figure 1:**
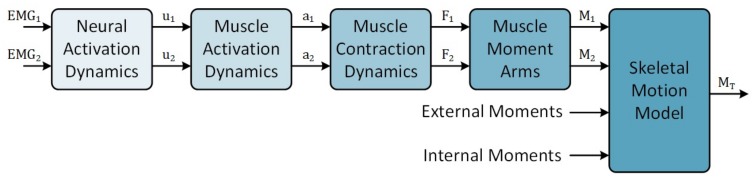
General muscle-level motion model framework illustrating the forward calculation of joint moment, $M_{T}$, from two antagonistically configured muscles (indicated by subscripts ${}_{1}$, ${}_{2}$). EMG is the raw EMG signal obtained from the muscle; *u* is neural activation generated from the EMG signal; *a* is the muscle activation signal; *F* is the force produced by each muscle; and *M* is the muscle torque generated about the joint of interest. This configuration represents a simplistic configuration of a muscle-level elbow motion estimation model.

In the absence of global solutions within the area of wearable elbow device control, a large number of motion models has been proposed [3,4,5,6,7,8,9]. These models can be arranged along a spectrum based on the level of decomposition of human motion properties. At one end of this spectrum, the dynamics of the human motor control system are treated as black-box models, such as in motion models built upon artificial neural networks [10], where inputs are mapped to outputs with limited internal knowledge of the properties. Towards the other end of the spectrum, the dynamics of the human motor control system are decomposed into sub-models, such as in the muscle-level model [3,9,11,12]. The general muscle-level model (Figure 1) describes internal force production as the summation of forces generated by motor units making up a given musculotendon unit. Since determining the force per motor unit requires invasive measurement, muscle-level models provide the opportunity to decompose motor control complexity while remaining in the realm of non-invasive sensing. Decomposing the dynamic behaviour of the human motor control system also provides opportunities to measure model parameters from the individual, such as electromechanical delay (EMD). This reduces the amount of optimization or configuration resources required to minimize estimation errors. Considering that wearable devices should be non-invasive, it would be ideal to use models that are decomposed to a level that involves only measurements of biological signals obtained from the surface of the human body. Current EMG signal measurement techniques allow detection of the electrical signals produced by a large area of muscle and support the use of muscle-level motion models [11,13,14,15].

Most of the muscle-level models for the estimation of the human motor control dynamics contain the following modules: neural activation dynamics, muscle activation dynamics, muscle contraction dynamics and skeletal dynamics [9,11,15,16]. Upon surveying these muscle-level models, many similarities can be found between the modules. However, the muscle activation dynamics module varies between these elbow motion models. Seven different muscle activation models have been identified as part of muscle-level models [9,13,14,17,18,19,20]. All of these models, with the exception of the one used in [20], form a portion of upper-limb motion estimation models. In the existing models, these muscle activation model parameters are optimized simultaneously with other parameters of the motion models to which they contribute. To the authors’ knowledge, no one has conducted an evaluation study specific to the performance and optimization of the muscle activation models.

The objective of this research is to determine which muscle activation model would be best suited for use in EMG-driven estimation of elbow motion. To meet this objective, an EMG-driven muscle-level motion model is developed to control the WearME elbow brace [21]. Elbow motion data collected from healthy subjects provide model input data for an optimization experiment. The experiment involves measuring the optimization performance of each muscle activation model under different elbow motion inputs. Comparison of the experimental results reveals limitations of the muscle activation models and opportunities for improving their performance.

## 2. Existing Muscle Activation Models

The neural activation model is the first transformation point for EMG signals in the general framework of musculoskeletal motion models. As explained by Buchanan et al., these models transform the normalized, rectified and filtered EMG signal into a bounded representation of the desired muscle activation using linear equations [11]. However, the presence of both linear and nonlinear isometric EMG-force relationships has been identified for different muscles [22,23]. As a result, muscle activation models are used to account for the nonlinear aspects of the EMG-to-muscle activation relationship.

Muscle activation models can be divided into two categories based on how the models describe the muscle activation dynamics. First, the muscle activation model is developed such that there is a linear and nonlinear component determined by a single parameter [13,14,17]. Manal et al. and Cavallaro et al. both define variations of a single-parameter exponential model [14,17]. The difference between these two models is whether the parameter is the base of the exponential component or part of the exponent. Manal and Buchanan developed a single-parameter piecewise curvilinear model, using a natural logarithm function [13]. One problem with these muscle activation models is that they have not been optimized separately from the elbow motion model in which they reside. This makes it difficult to determine the specific performance of these muscle activation models.

The other avenue for designing muscle activation models is based on the concept that there is an EMD for both muscle activation and muscle deactivation, making them two-parameter models [9,18,19,20]. The output of these models varies based on whether the muscle is in a state of muscle activation or muscle deactivation. Rengifo et al. and Thelen incorporate this property of muscular control through piecewise differential functions in which the regions are based on the ratio of neural activation to muscle activation [18,20]. Chadwick et al. propose two different functions in which the time delay relationship is incorporated into a non-piecewise first-order differential equation [9,19]. Commonly, the activation and deactivation time constants are chosen at fixed values based on previous studies of control of human muscle [9,18,19,20]. Winters describes the process by which muscle activation and deactivation are related to chemical processing within the muscle [24]. Since the availability of chemicals may vary with respect to time, it is plausible that muscle activation and deactivation delays also vary. Recently, Downey et al. offered some evidence to support this notion by showing changes in EMD in response to fatigue induced in the quadriceps femoris muscle [25]. However, to maintain consistency with previous results, the EMD values in the experiment presented here are chosen as constants. The functional definitions of the muscle activation models are included in Table 1.

## 3. Methods

Evaluating the muscle activation models for control of a wearable mechatronic elbow brace requires developing an elbow motion model to host the various muscle activation models. However, wearable mechatronic devices are driven using embedded computer systems, for which computational resources are limited. Kiguchi and Hayashi were able to achieve a very high accuracy in elbow moment estimation, but note that their analysis would not be suitable for real-time control [8]. While studying muscle synergies during throwing, Ruiz et al. detail optimization time measurements that range between 1 and 12 h using a desktop computer system for datasets whose length is seconds [12]. The architecture of the developed elbow model is influenced heavily by this computational limitation.

**Figure 2:**
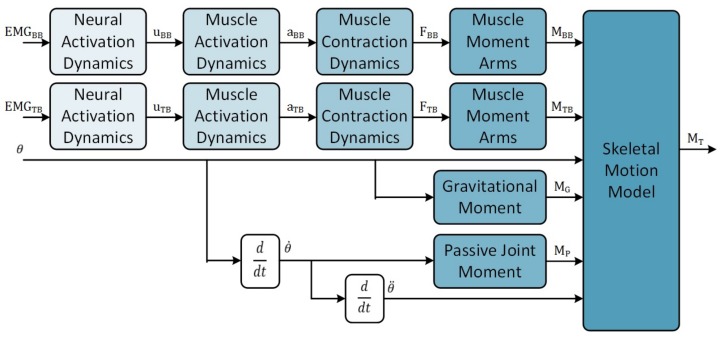
The developed elbow motion model uses EMG signals of the biceps brachii, $EMG_{BB}$, and the triceps brachii muscles, $EMG_{TB}$, combined with the current elbow angle to estimate the total joint torque of the elbow, $M_{T}$. The two muscle torques are configured antagonistically within the skeletal motion model. $M_{G}$ is the moment caused by gravitational forces; $M_{P}$ is the passive joint torque; and θ, $\dot{\theta }$ and $\ddot{\theta }$ are the joint position, angular velocity and angular acceleration, respectively.

In this paper, two architectural design choices were made in order to address this limitation. First, the general muscle-level model framework (Figure 1) has been adopted due to the ability to use subject-specific parameters and reduce the computational resources required for model optimization. Second, the number of inputs has been limited as the size of the model increases with an increased number of inputs. Only two EMG channels, one each from the biceps brachii and triceps brachii muscles, together with the elbow joint position are used as inputs to the model to fulfil this requirement. The implementation of the elbow motion model (Figure 2) is described in the following sections.

### 3.1. EMG Processing and Neural Activation Model

The first step in the process for providing motion estimates is to transform the EMG signals into neural activation signals. This action is performed by processing the raw EMG signals, e(t), and then producing the neural activation, u(t), through a model that describes the neural activation dynamics. Processing the raw EMG signals involves removing the DC offset, filtering to remove noise, rectification and, finally, normalization. Normalization is completed through the following equation using values obtained from the subject during a maximum voluntary contraction (MVC) of the desired muscle:

(1) $$e_{norm}\left(t\right)=\frac{e\left(t\right)-EMG_{min}}{EMG_{max}-EMG_{min}}$$

where $e_{norm}\left(t\right)$ is the normalized EMG signal and $EMG_{max}$ and $EMG_{min}$ are the maximum and minimum values collected from the EMG signal during the MVC. The processed EMG signal is a normalized signal, which intends to maintain a bounded response of the EMG signal. However, further translation is required to account for linear and nonlinear characteristics in the EMG-force relationships.

It has been suggested that a fourth-order low-pass Butterworth filter (zero-phase when possible) should be used as a final step in converting the EMG signal into the neural activation signal [11]. Cut-off frequencies for these filters range from 2–10 Hz in the literature [11,13,14,26,27,28,29]. Based on frequency spectrum analysis of the collected EMG signals, the implemented neural activation model consisted of a fourth-order low-pass Butterworth filter with a cut-off frequency, $f_{c}$, of 3 Hz.

### 3.2. Muscle Activation Models

Muscle activation dynamics describe the relationship between the neural activation generated from the central nervous system and the mechanical activation, a(t), of the muscle actuator [17]. This component of the elbow model (Figure 2) is a single-input single-output system whose parameters must be optimized to provide the appropriate degree of nonlinearity. The seven muscle activation models described in Table 1 were used to define this component in the elbow model.

### 3.3. Muscle Contraction Model

**Figure 3:**
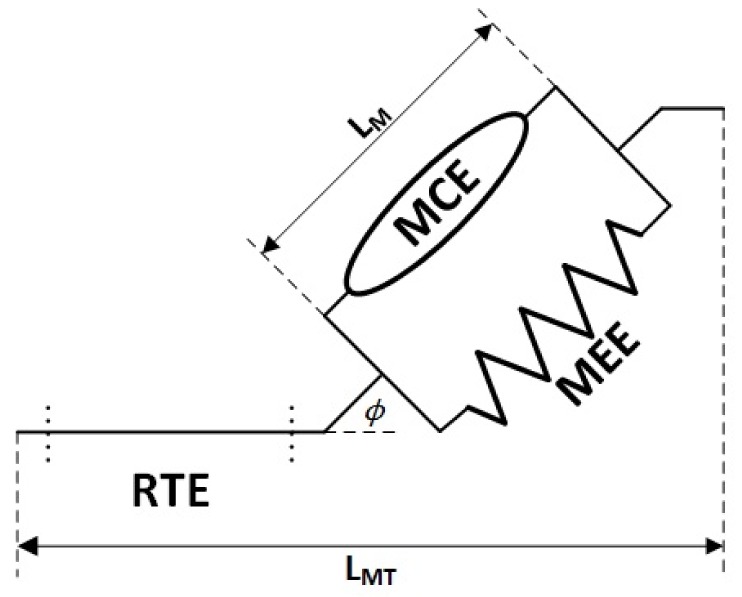
A Hill-type muscle model defines the dynamic contractile properties of the musculotendon unit. This model is made up of the following three components: muscle contractile element (MCE), muscle elastic element (MEE) and rigid tendon element (RTE). The MCE models the active forces of the muscle unit while the MEE models the elastic properties of the muscle unit. The RTE is modelled as a rigid body that transfers muscle force directly to attachment points on the bone. $L_{MT}$ is the length of the musculotendon unit; $L_{M}$ is the length of the muscle unit; and ϕ is the pennation angle.

The muscle contraction model (Figure 3) defines both the active and the passive forces generated by the musculotendon unit. To describe this dynamic behaviour, a Hill-type muscle model is commonly used [3,9,11,13,14,18,19,30]. The tendon element, typically modelled as an elastic element, is modelled as a rigid tendon element (RTE) in this muscle contraction model. This modification was made as Millard et al. have shown that an RTE offers an increase in simulation speed and similar force estimation errors when compared to other elastic tendon element models used with a sub-maximally-activated Hill-based muscle model [31]. The output of the muscle contractile element (MCE), $F_{MCE}$ (typically defined as $F_{active}$), is derived from the mechanical activation, the maximum isometric force ($F_{max}$), the isometric force-length relationship ($F_{fl}\left(L_{M}\right)$) and the force-velocity relationship ($F_{fv}\left({\dot{L}}_{M}\right)$) of the muscle, as follows:

(2) $$F_{MCE}=aF_{max}F_{fl}\left(L_{M}\right)F_{fv}\left({\dot{L}}_{m}\right)$$

The active force potential of the muscle is dependent on the length of the muscle as described by the following force-length relationship:

(3) $$F_{fl}\left(L_{M}\right)=e^{{\left(-\frac{L_{M}-L_{M_{o}}}{WL_{M_{o}}}\right)}^{2}}$$

where $L_{M}$ is the length of the muscle (Figure 3), $L_{M_{o}}$ is the muscle length at which the the most force is expressed (optimal length) and *W* is a shaping factor. The force-velocity relationship describes the difference in force potential as a function of velocity, which varies depending on whether the muscle is concentrically (${\dot{L}}_{M}\leq 0$) or eccentrically (${\dot{L}}_{M}>0$) contracting [9]:

(4) $$F_{fv}\left({\dot{L}}_{M}\right)=\left\{\begin{array}{cc} \frac{{\dot{L}}_{M_{max}}+{\dot{L}}_{M}}{{\dot{L}}_{M_{max}}-\frac{{\dot{L}}_{M}}{A}}, & {\dot{L}}_{M}\leq 0\\ \frac{g_{max}*{\dot{L}}_{M}+c_{d}}{{\dot{L}}_{M}+c_{d}}, & {\dot{L}}_{M}>0 \end{array}\right.$$

where ${\dot{L}}_{M}$ is the velocity of the muscle, ${\dot{L}}_{M_{max}}$ is the maximum shortening velocity ($10\cdot {\dot{L}}_{M_{o}}$ meters per second), $g_{max}$ is the maximal normalized eccentric force and *A* is the Hill curve shaping parameter. Chadwick et al. define $c_{d}$ as a constant used to ensure a continuous first derivative at ${\dot{L}}_{M}=0$ [9]:

(5) $$c_{d}=\frac{{\dot{L}}_{M_{max}}*A*\left(g_{max}-1\right)}{A+1}$$

The muscle elastic element (MEE) represents the passive force output of the muscle fibres. This structure exhibits elastic properties that are dependent on muscle length. In this Hill-type model, the MEE is modelled as a nonlinear spring [9], as follows:

(6) $$F_{MEE}\left(L_{M}\right)=\left\{\begin{array}{cc} k_{1}\left(L_{M}-L_{S}\right), & L_{M}\leq L_{S}\\ k_{1}\left(L_{M}-L_{S}\right)\\ +k_{2}{\left(L_{M}-L_{S}\right)}^{2}, & L_{M}>L_{S} \end{array}\right.$$

where $k_{1}$ and $k_{2}$ are stiffness coefficients and $L_{S}$ is the muscle slack length. $L_{S}$ is chosen to be the $L_{M_{o}}$ except in situations resulting in high passive forces [9]. In this elbow model, the muscle contraction module is modelled as an equilibrium musculotendon model [31]. Using this formulation of the musculotendon unit, the forces generated by the muscle and the tendon are in equilibrium, as follows:

(7) $$F_{RTE}=\left(F_{MCE}+F_{MEE}\right)\cos\left(\phi \right)$$

where ϕ is the pennation angle of the muscle fibres. Pennation angle is calculated using a constant volume assumption [9], as follows:

(8) $$\phi =\frac{L_{M_{o}}\sin\left(\phi_{o}\right)}{L_{M}}$$

where $\phi_{o}$ is the pennation angle at the optimal muscle length. Combining Equations (7) and (8), $F_{RTE}$ becomes the output of the muscle contraction model.

In order to customize the muscle contraction model to each subject, OpenSim (National Center for Simulation in Rehabilitation Research, California, U.S.A.) was used to derive estimates of the model parameters. First, the OpenSim Upper Lower Body Model was scaled based on limb lengths measured from each subject [32]. Next, optimal muscle fibre length ($L_{M_{o}}$) and pennation angle at optimal fibre length ($\phi_{o}$) values were taken for the biceps brachii long head and triceps brachii long head muscles. Since this elbow model is developed as a two-muscle system, the maximum isometric force ($F_{max}$) of the major flexor muscle units (biceps brachii short head, biceps brachii long head, brachialis, brachioradialis and pronator teres) and of the major extensor muscle units (triceps brachii lateral head, triceps brachii long head, triceps brachii medial head and anconeus) were summed to represent the maximum isometric force of each of the flexor and extensor muscle model, respectively. This design choice was made to allow for a simpler two-muscle elbow model that could be driven by two surface EMG signals while maintaining approximately similar total muscle forces, as would be generated by the separate muscle units. Constant values taken from OpenSim are listed in Table 2. Finally, data defining the relationship between muscle unit length ($L_{M}$) and elbow joint angle (θ) and between musculotendon unit length ($L_{MT}$) and elbow joint angle were exported to MATLAB (MathWorks, USA) in order to define muscle length as a function of joint angle using the curve fitting function *fit*.

### 3.4. Skeletal Motion Model

The skeletal motion model describes the relationship between all sources of joint motion. To determine moments generated by the muscles, the moment arms of the musculotendon units must be determined. These moment arms, *r*, are determined by differentiating the muscle length with respect to the joint angle for each muscle [9], as follows:

(9) $$r=\frac{d}{d\theta }L_{MT}\left(\theta \right)$$

Height and weight measurements combined with the proportionality equations defined by Winter provide estimates of the gravitational forces acting on the lower arm [33]. A simple cylindrical model of the lower arm provides an inertial estimate for this skeletal model. The passive joint torque equation is modified from the equation proposed by Chadwick et al. [9] to include only damping, as follows:

(10) $$M_{p}=-b\dot{q}$$

where *b* is a damping coefficient. Chadwick et al.’s equation includes a stiffness model that defines that stiffness forces push towards the middle of the joint’s range of motion. However, this chosen position and stiffness force distribution may vary based on the individual, the joint position limits and the joint structure. Since joint stiffness was not profiled for the participants, they had healthy elbow joints, and they completed motions that were restricted within their joint limits; the passive joint torque equation has been simplified.

Finally, these quantities are combined into an equation of motion by summation of joint moments:

(11) $$M_{joint}=I\ddot{\theta }+M_{p}+M_{g}+\sum_{i=1}^{n}r_{i}F_{RTE_{i}}$$

where $M_{joint}$ is the total resulting joint torque, *I* is the inertial mass, $M_{g}$ is the moment due to gravitational forces, *i* denotes the *i*-th muscle and *n* is the number of muscles contributing to joint motion. The resulting output of the model is the total torque about the elbow joint.

### 3.5. Performance Metrics

Performance metrics allow the comparison of expected and measured outputs of the models. The most commonly-measured performance metric in the elbow model literature is accuracy [7,8,10,12,15,16,34,35,36,37]. Accuracy is a measurement of the similarity between an estimated elbow motion parameter and a measured elbow motion parameter. Indeed, this is an important aspect to determine performance, but many of these accuracy measurements were conducted on computer systems that do not match the computational resources available with the embedded control systems of wearable devices. Improving accuracy comes at the cost of computational resources, which ultimately can be measured in time. Adoption of these devices by users or clinicians will be impossible if each 10-min usage could take hours to optimize. To the authors’ knowledge, only one study, by Millard et al., contains a comparison of accuracy and computational resource usage [31]. However, their study focused on comparing three different musculotendon models using a constant muscle activation model. The study presented here follows a similar process towards identifying accuracy and computational resource usage and supplements the previous work by evaluating various muscle activation models while keeping the musculotendon model constant. Using the metric suite presented in Table 3, the performance of the muscle activation models can be evaluated.

## 4. Experimental Evaluation

Based on the desire to use motion models for control of a wearable elbow device, the experiment is developed as a model optimization scenario. The main purpose of this experiment was to evaluate and compare the performance of the muscle activation models using EMG inputs collected during various elbow motions. Six healthy subjects, three males and three females, with ages ranging from 21–26 and no known injury or disease affecting the upper-limb participated in the experiment. Data collected from these subjects were used as the inputs for the model optimization. The specific goal of the optimization was to produce the highest accuracy in estimation for each muscle activation model while quantifying the computational resources required to achieve these results. All subjects gave their informed consent before participating in this study. Ethics approval was granted for this experiment from the Human Research Ethics Board at Western University (Reference No. 105717).

### 4.1. Data Collection and Processing

**Figure 4:**
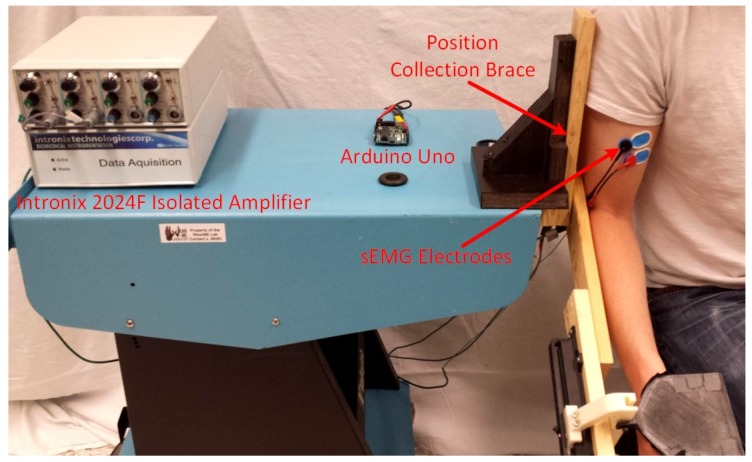
The data collection setup consists of the Intronix 2024F Isolated Amplifier and the elbow position collection brace. Bipolar surface EMG electrodes used by the amplifier to collect the electrical muscle activity. One pair of electrodes was placed over the skin covering each of the biceps brachii and triceps brachii muscles, while a single reference electrode was placed near the olecranon. Subjects were connected to the collection brace using Velcro straps (not shown) such that their elbow axis aligned with the rotational axis of the brace. An Arduino Uno was used to collect encoder data from the collection brace and stream it to a desktop computer.

The elbow model relies on both EMG and motion data from subjects. In this experiment, EMG data from the biceps brachii and triceps brachii (long head) muscles and position data from the elbow joint were collected during elbow motion. The skin over the muscle of interest was cleaned using alcohol, and a surface EMG electrode was placed on the skin and connected to an Intronix 2024F Isolated Amplifier (Intronix Technologies Corporation, Bolton, ON, Canada), which sampled the EMG signals at a frequency of 4000 Hz. First, the subjects were asked to complete MVCs of both the biceps brachii and triceps brachii muscles. Next, subjects were seated next to and strapped into an elbow position collection brace mounted to a table-top surface as shown in Figure 4. This device housed an optical encoder aligned with the elbow joint axis of rotation and sampled the elbow position using an Arduino Uno sampling at a frequency of 100 Hz. Subjects were then asked to complete one repetition of a full range elbow flexion-extension motion followed by four other elbow motions completed for three repetitions each (see Table 4).

The EMG and position signals were processed before being used in the optimization procedure. Raw EMG signals were bandpass filtered (Butterworth second order 20–300 Hz), rectified and normalized to the values obtained from the MVC datasets. Position data were upsampled from 100 Hz to 4000 Hz and low-pass filtered (Butterworth fourth order 3-Hz cut-off frequency) to remove noise from the signal. Finally, the EMG and position datasets were synchronized based on the timestamps given during collection.

### 4.2. Optimization Procedure

**Figure 5:**
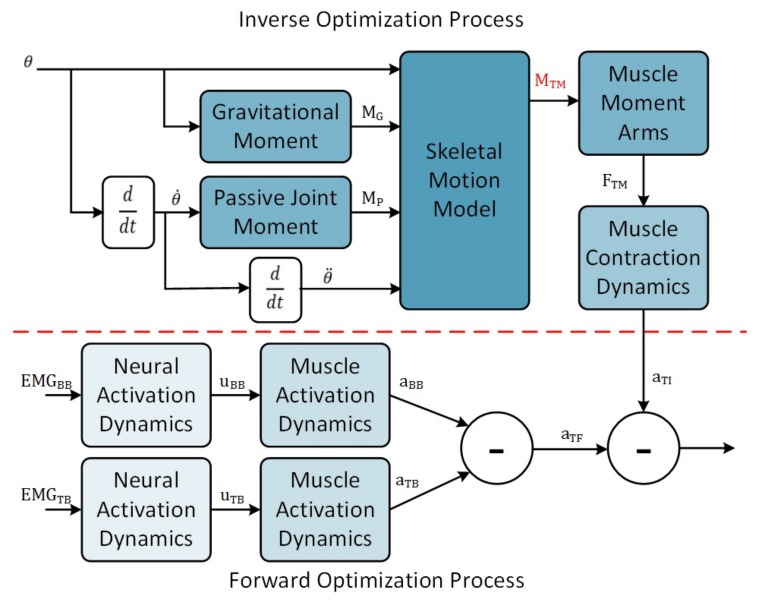
The inverse optimization procedure determines the optimal total muscle torque, $M_{TM}$, to minimize the elbow joint moment. Then, the total muscle activation, $a_{TI}$, is derived from the total muscle torque. The forward optimization process minimizes the error in muscle activation signals by determining the optimal parameters values for each muscle activation model. These two processes (separated by the red dotted line) are completed sequentially for each dataset. $F_{TM}$ is the total muscle force; $a_{TI}$ is the inversely-derived total muscle activation; and $a_{TF}$ is the forward-derived total muscle activation.

Optimizing the muscle activation models was accomplished using both an inverse and forward optimization for each recorded motion. These procedures were completed using a desktop computer system with an Intel i7-4770 quad-core processor and 16 GB of DDR3 RAM running the Windows 10 operating system. The inverse optimization began by finding the total muscle torque that minimizes Equation (11) at each position along the trajectory of the recorded motion. Next, the muscle activations were determined from the resulting total muscle torque. However, the relationship between the activations of the two muscles are dynamic and unknown, leading to an indeterminant system. To work around this limitation, a single musculotendon unit, sharing the properties of the biceps brachii muscle, was used. The muscle activation was inversely derived from the optimized muscle moment as shown in Figure 5.

The forward optimization was completed in order to optimize the parameters of each muscle activation model. Each EMG channel, two in this case, has an associated muscle activation model where the sum of the activation forms the total muscle activation (Figure 5). A similar minimization process as the one described by Buchanan et al. is used [11]. However, the procedure used here minimizes the squared muscle activation error (the first Equation in Table 3) as opposed to minimizing of the squared joint moment error. Both optimizations were completed using a constrained minimization implemented in MATLAB using the fmincon function. For the inverse optimization, only a single parameter, the total muscle torque, was used as an optimization variable. The forward optimization procedure required optimization of either one parameter (Model 1 and Model 2) or two parameters (Models 3–7) depending on the muscle model used. Parameter ranges for Model 1 ($A_{1}$:−3–0) and Model 2 ($A_{1}$:0.05–1) were taken from the literature [14,17]. For Model 3, it was decided to optimize two model parameters simultaneously instead of the nested minimization used by Manal et al. [13]. The range of the $A_{1}$ parameter was listed in [13] (0.0001–0.12), and the $A_{2}$ parameter range was chosen by experimenting with the limits of the range (0.01–$10^{11}$). Models 4–7 did not optimize activation and deactivation parameters in their studies [9,18,19,20]. Time constants of the elbow muscles have been listed as 5–40 ms and 20–70 ms in the literature for muscle activation and deactivation, respectively [38,39]. As a result, the optimization range for these values was chosen to be 0–70 ms. The parameter ranges for both forward and inverse optimizations are shown in Table 2. Following each forward optimization, the optimized parameters were used in conjunction with the elbow model to estimate the elbow joint moment.

During the experiment, the total muscle activation error, total muscle torque error and data point optimization time metrics were measured to facilitate comparison of accuracy and computational time. The processor usage percentage and program space were monitored across all forward optimization trials in order to establish an average computational demand. A statistical analysis was performed using Kruskal–Wallis H tests since the collected data exhibited non-normal distributions. The Kruskal–Wallis H tests were conducted with the model, motion and number of optimization parameters as fixed factors and the total muscle activation error, the total muscle torque error and the data point optimization time metrics as the dependent variables. Thirty-one pair-wise Kruskal–Wallis tests were conducted using IBM SPSS Software (Version 24, IBM Corporation, Armonk, NY, USA) based on 7 muscle activation model groups, 5 motion groups, and 2 optimization parameter groups. Statistical significance was determined using an α value of 0.05.

## 5. Results

During the experiment, 78 elbow motion datasets were collected (13 per subject). These datasets were used in the inverse optimization procedure to generate 78 muscle activation trajectories used as the controls. For each of these control trajectories, seven muscle activation trajectories, one for each muscle activation model, were optimized using the forward optimization procedure. This resulted in 546 muscle activation trajectories to be used in the evaluation. During the forward optimization procedure, the performance metrics, described in Section 3.5, were measured. Average results for total muscle activation error, total muscle torque error and data point optimization time grouped by muscle activation model, subject motion and number of optimization parameters are listed in Table 5.

**Figure 6:**
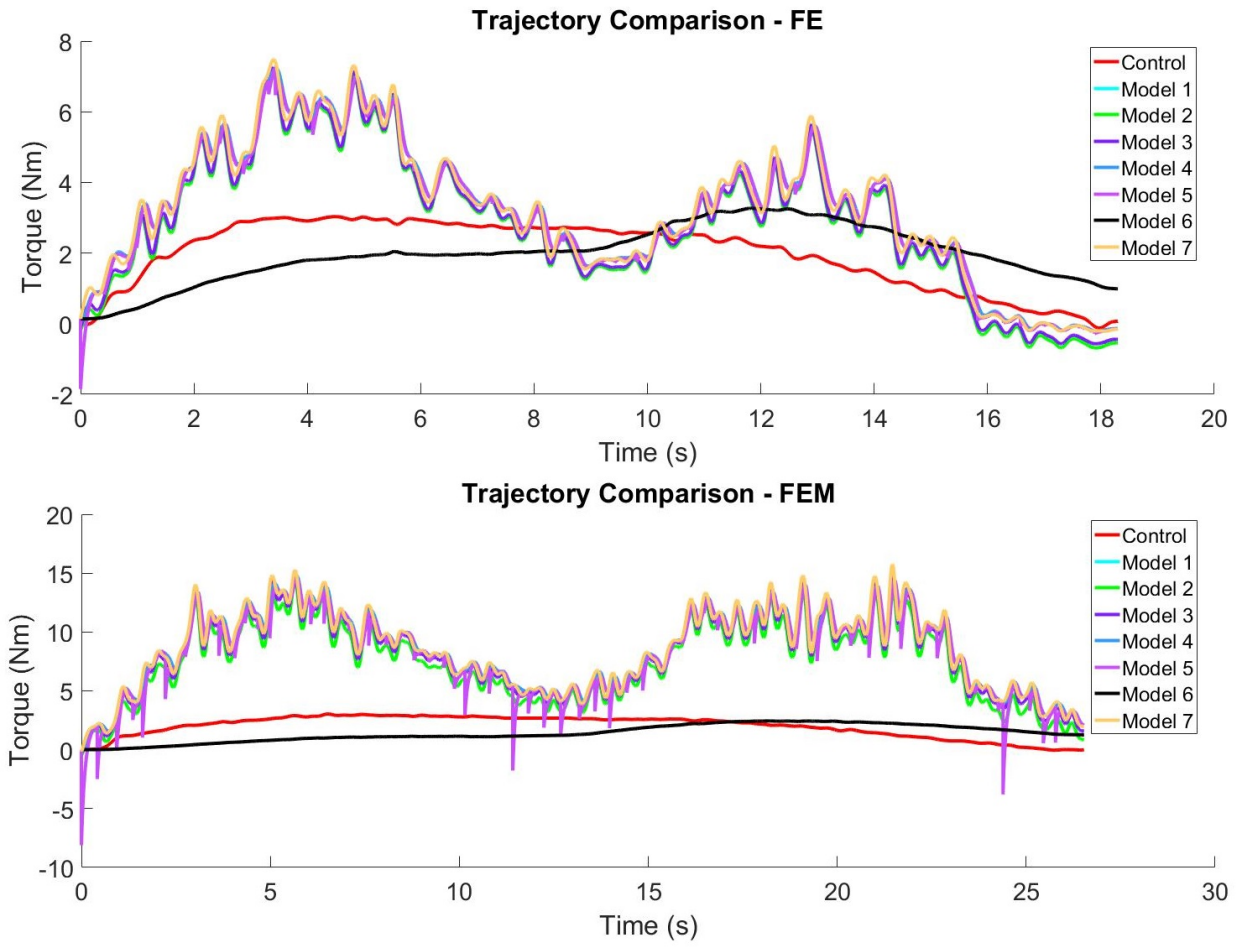
The variance in muscle torque estimates with respect to the optimized torque is shown for both the FE (top graph) and FEM (bottom graph) motions of a representative subject.

An average total muscle activation error of 0.00082 ± 0.00108, across datasets, was determined from the experiment. Considering that the total muscle activation is a normalized value of two opposing muscles, whose summed value could range between −1 and 1, this average error represents a percentage of 0.041% of this range. Total muscle torque errors, derived from the optimized muscle activation model trajectories, resulted in a 2.09 ± 1.39 Nm error across all datasets. An average torque error of 3.4–4.2 Nm shown in the motion model developed by Cavallaro et al. suggests that the torque errors determined in this experiment are consistent with those in the literature. Figure 6 shows examples of the FE (top graph) and FEM (bottom graph) control and optimized trajectories of each model.

The data point optimization time of the forward optimization procedure was 317 ± 416 μs on average. This large standard deviation is due to relatively large standard deviations compared to averages in the data point optimization time of the muscle activation Models 4, 5 and 7 (see Table 5, Column 4). The longest optimization time during the forward optimization procedure was 616 s. In terms of processing requirements, the average processor usage percentage was measured to be 100.0013% of a possible 400% available quad-core processing time, while requiring an average of 94.91 MB of program space to execute the forward optimization trials. It should be noted that the algorithms were not implemented as a solution that fully utilizes multi-core processing. The duration of one inverse optimization process ranged approximately from 1–4 h.

The statistical analysis of these results indicated many significant differences between models, motions and the number of optimization parameters. Total muscle activation error showed statistically-significant differences between models, motions, and number of parameters. Both Model 1 and Model 2 showed statistically smaller muscle activation errors than Model 5 (*p* value = 0.027) and Model 7 (*p* value = 0.022). Regarding motions, the FES motion produced statistically smaller errors than the EF120 (*p* value = 0.013), FEM (*p* value = 0.003) and FEP (*p* value = 0.012) motions. The number of optimization parameters showed a statistical difference (*p* value = 0.008) between one-parameter models (Model 1 and Model 2) and two-parameter models (Models 3–7), with one-parameter models exhibiting smaller errors. However, this statistical significance between the number of parameters did not carry through to the total muscle torque error.

**Figure 7:**
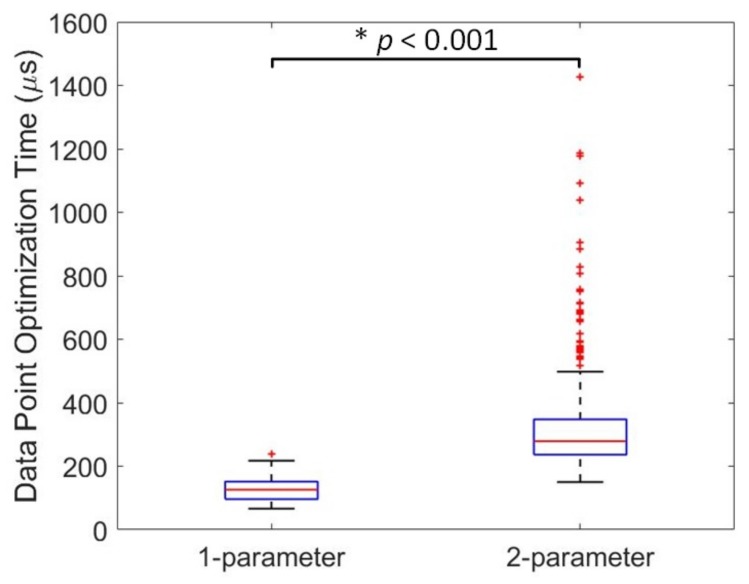
Data point optimization times vary with a statistically-significant difference with respect to the number of optimization parameters of each muscle activation model.

Statistical differences were observed between the models and the motions for the total muscle torque error metric. Model 6 showed a statistically-significant lower muscle torque error compared to Model 5 (*p* value = 0.039) and Model 7 (*p* value = 0.049), but was not statistically different from the other models in this metric. The total muscle torque error for the FES motion was statistically lower than the FEM (*p* value = 0.004) and FEP (*p* value = 0.007) motions, similar to the total muscle activation error, but was not statistically different than the FE or EF120 motions. Data point optimization time exhibited differences between the models and the number of optimization parameters, but not between motions. All models showed statically-significant differences in data point optimization time (*p* value < 0.001), except between Model 5 and Model 6. There was also a statistically-significant difference in data point optimization time between muscle activation models with one- and two-optimization parameters (*p* value < 0.001), as shown in Figure 7.

## 6. Discussion

The main contribution of this research is the evaluation of muscle activation models regarding their ability to estimate elbow motion. The results reveal that the muscle torque estimation error generated using Model 6 is on average lower (1.67 ± 0.74 Nm) than all other muscle activation models considered in the experiment, but only statistically different from Model 5 (2.19 ± 1.47) and Model 7 (2.19 ± 1.50). This result is emphasized in the muscle torque trajectories of Figure 6, where Model 6 can be distinguished from the other models. However, it can also be seen that none of the models follow the shape of the control trajectory particularly well. The discrepancy between the control and optimized trajectory shapes may be due to modelling errors in either the muscle activation models, the other components of the model or unmodelled dynamics, but the origin of these errors is still being investigated.

Compared to elbow joint torque estimation error of the model proposed by Cavallaro et al. (average error: 3.4–4.2 Nm), an improvement in torque estimation was seen, regardless of muscle activation model (average error: 1.67–2.19 Nm). It is important to note that the results presented here considered the error between the control and optimized muscle torque values, not the control and optimized joint torque values, as was completed in Cavallaro’s model. However, the same datasets were used to determine the control and optimized torque contributions that did not stem from the musculature, meaning that the error in muscle torque represents the error in the joint torque. None of the other models considered in this study measured torque estimation errors. Although an improvement in estimation error of this elbow model has been shown over Cavallaro’s model, even a torque error of a few Nm could cause excess strain on soft tissues of the human body or cause the user to strain excessively thinking the device will provide appropriate assistance. Both of these scenarios can lead to further injury of the user and require improvements to the estimation accuracy of the models.

In general, the results show that none of the models are good enough for accurate motion and torque control of a wearable mechatronic device, as even the lowest errors are too high to ensure safety. These errors are likely a result of unmodelled dynamic properties. For example, variation in muscle stiffness has not been considered in any of these models. Furthermore, an assumption was made that the biceps and the triceps contained all of the force potential, including that of all major flexors and extensors of the elbow, which we know not to be true. Furthermore, the fact that the tasks are being performed at very slow speed likely has an effect on the smoothness of the motion. Finally, it is possible that incorrect assumptions have been made, or that there are components of the model between the neural and the muscle activation dynamics that are not accounted for. EMG activation is simply not fully understood yet, so further work in this area can lead to improvements in the results.

On the other hand, there are other sources of error that should not necessarily be considered for the control of wearable devices. For example, electrode placement will have an effect on the signals collected, and it is possible to develop new techniques to find muscle locations more accurately in order to decrease variability. However, doing this will not properly reflect real scenarios in which the therapist or the patient is putting on the device, justifying the need for more robust models that account for this type of variability. Similarly, significant subject variability was observed in the collected data. The variability was more pronounced in patterns of muscle co-contraction, especially at points where the motion changed direction, when starting or when stopping. These variations may be caused by factors such as stress, level of motivation, muscle tone, level of fatigue, and experience with performing similar motions. All of these factors would be very difficult to measure in a real case scenario and even more difficult to determine their effect on the collected data.

One method to improve accuracy is to use a larger number of optimization parameters as this increases the number of possible solutions that can fit the data. However, increasing accuracy using this method leads to increased computational demand. Buchanan et al. describe that another problem with this approach is the overfitting of the data, which leads to a reduction in the predictive power of the model [11]. However, one solution to increasing accuracy while decreasing computational demand comes from increasing the complexity of biological models. By studying and understanding the mechanisms that generate movement more thoroughly, these biomechanical models can evolve to include components that account for more of the dynamic properties of the human motor control system. Increasing the complexity of the models will generate more parameters to optimize, which as stated above, could increase computational demand. However, if the complexity is modelled such that the parameters can be measured from the subject, the computational demand for the optimization of the model could be reduced.

The statistical analysis revealed other significant findings, namely that muscle torque and muscle activation errors differ by motion and that data point optimization time differs by model and number of optimization parameters. In terms of muscle torque and muscle activation estimation errors, the differences found between motions suggest that the muscle activation models may be suited better for certain motions than others. For example, the FES motion differs significantly from the FEM and FEP motions with respect to torque estimation error due to these latter ones having unmodelled dynamics, such as the added mass or discontinuities in trajectory smoothness, respectively. Both added masses and pauses in motion are common in rehabilitation scenarios and activities of daily living and, therefore, must be accounted for within these models. Another possibility for FES being better than the other motions is that the starting torque is higher due to the motion starting at an increased elbow angle. This behaviour matches models that reflect a torque pattern that begins at a larger positive value, hence matching the torque estimation observed from most models (except Model 6), as shown in Figure 6.

Differences in data point optimization time emphasize that some models may be better choices when optimization is required and computational resources are limited. Based on the results, the models can be ranked from smallest to largest data point optimization time as follows: Model 1, Model 2, Model 3, Model 5 or 6, Model 7 and Model 4. Therefore, Model 1 would be ideal if optimization time is the highest priority, as it produces a torque error not statistically different from the other models while exhibiting the lowest data point optimization time. Finally, models with two optimization parameters had a significantly longer data point optimization time and larger total muscle activation error than models with one optimization parameter. However, there does not exist a significant difference in torque error between one-parameter and two-parameter models.

Ultimately, this study highlights the trade-off between the accuracy of the model and the computational expense. While estimation accuracy provides a means to determine theoretical feasibility, computational demand provides a quantification of concrete feasibility regarding the use of these models in wearable assistive devices. It is common to develop and study these motion models using computer systems that can execute more than one million instruction per second and in situations where optimization time is infinite. In the presented experiment, the forward optimization took up to 10.3 min on such a computer system. However, even this length of optimization may be too long to consider using this model in a wearable device that may need to execute only a few movements at a time. Furthermore, moving this computational task to an embedded computer system, where the instructional velocity may be far less than the one used in this experiment, would only increase the optimization time to prohibitive levels. Accompanying this with the fact that optimizations may need to be conducted at each usage, or for each motion, due to variability in biological signals and environmental factors, a major limitation for adoption of these models and the devices that require them lies in the computational expense.

## 7. Conclusions

The caution surrounding integration of human and machine is founded on both the utility of the action and the assured safety of both systems. Modelling human motion is a means towards increasing the utility and safety of wearable assistive technologies, but is dependent on the discovery of model limitations. Using a developed elbow motion model, seven muscle activation models were evaluated in their ability to estimate elbow motion. The experimental results show that torque errors ranged from 1.67–2.19 Nm and average optimization time per data point ranged from 99.75–766.40 μs. A statistical evaluation indicated many significant differences between the models, the motions preformed by the users and in the number of optimization parameters. Although Model 6 showed the lowest torque estimation error, the results were not statistically different from all other models. In any case, differences in model performance based on variation in elbow motion suggest that improvements are needed to increase the robustness of these models. The results from these trials emphasize the need for developing better models, as existing torque errors are too high for their safe implementation into a wearable mechatronic device. Possible sources of error have been identified and include unmodelled dynamic properties and simplifications in the number of factors considered by the models. Future work includes further evaluation of motion models, and their components, during human-machine interactions in order to find solutions that increase the utility and safety of wearable assistive devices and their users.

## Figures and Tables

**Table 1:** Functional definitions for the seven muscle activation models considered in this study.

Model ID	First Author [Reference]	Model Equations *
Model 1	Manal [17]	$a\left(t\right)=\frac{e^{A_{1}u\left(t\right)}-1}{e^{A_{1}}-1}$
Model 2	Cavallaro [14]	$a\left(t\right)=\frac{A_{1}^{u(t)}-1}{A_{1}-1}$
Model 3	Manal [13]	$u_{0}=0.3085-A_{1}\cos\left(45^{{}^{\circ}}\right)$ $a_{0}=0.3085+A_{1}\sin\left(45^{{}^{\circ}}\right)$ $m=\frac{a_{0}-1}{u_{0}-1}$ c=1−m $\beta =\frac{e^{a_{0}/A_{2}}-1}{u_{0}}$ $$a\left(t\right)=\left\{\begin{array}{cc} A_{2}\ln\left(\beta u\left(t\right)+1\right), & 0\leq u\left(t\right)\leq u_{0}\\ mu(t)+c, & u_{0}\leq u\left(t\right)\leq 1 \end{array}\right.$$
Model 4	Chadwick [9]	$\dot{a}\left(t\right)=\left(\frac{u(t)}{A_{1}}+\frac{1-u(t)}{A_{2}}\right)\left(u\left(t\right)-a\left(t\right)\right)$
Model 5	Rengifo [18]	$$\dot{a}\left(t\right)=\left\{\begin{array}{cc} -\frac{a(t)}{A_{1}}+\frac{u(t)}{A_{1}}, & u(t)\geq a(t)\\ -\frac{a(t)}{A_{2}}+\frac{u(t)}{A_{2}}, & u(t)\;<\;a(t) \end{array}\right.$$
Model 6	Chadwick [19]	$\dot{a}\left(t\right)=\left(A_{1}u\left(t\right)+A_{2}\right)\left(u\left(t\right)-a\left(t\right)\right)$
Model 7	Thelen [20]	$\dot{a}\left(t\right)=\frac{u(t)-a(t)}{T_{a}}$ $$T_{a}=\left\{\begin{array}{cc} A_{1}\left(0.5+1.5a\left(t\right)\right), & u(t)>a(t)\\ A_{2}/\left(0.5+1.5a\left(t\right)\right), & u(t)\leq a(t) \end{array}\right.$$

* Optimization parameters of the muscle activation models are denoted *A*_1_ and *A*_2_; *u*(*t*) is the neural activation signal; *a*(*t*) is the muscle activation signal; *u*_0_ and *a*_0_ are the transition point between the linear and non-linear portions of the *u*(*t*)–*a*(*t*) relationship; *t* is time, *β*, *m* and *c* are constants; and *T_a_* is an activation time constant.

**Table 2:** Description and source information for elbow model constants.

Parameter Symbol	Parameter Source	Parameter Values (Units)	
**EMG Processing and Neural Activation Model**			
$f_{c}$	Analysis, [2,27]	3 Hz	
**Muscle Contraction Model**			
		Biceps Brachii (Long Head)	Triceps Brachii (Long Head)
$F_{max}$	OpenSim	2874.67 N	2397.12 N
$L_{M_{o}}$	OpenSim	0.1106–0.1361 m	0.1236–0.1681 m
$\phi_{o}$	OpenSim	0 rad	0.2094 rad
*W*	[9]	0.56	0.56
${\dot{L}}_{M_{max}}$	[9]	1.106–1.361 m/s	1.236–1.681 m/s
*A*	[9]	0.25	0.25
$g_{max}$	[9]	1.5	1.5
$L_{slack}$	[9,19]	0.1659–0.2042 m	0.1854–0.2522 m
**Skeletal Motion Model**			
*b*	[9]	1 Nms/rad	

**Table 3:** Performance metrics suite to compare between accuracy and computational requirements.

Name	Description	Equation
Total muscle activation error	The root-mean square of the squared difference between the forward-derived total muscle activation ($a_{TF}$) and the inversely-derived total muscle activation ($a_{TI}$). $N_{s}$ is the number of samples.	$$e_{a}=\sqrt{\frac{1}{N_{s}}\sum_{i=1}^{N_{s}}{\left({\left(a_{TI_{i}}-a_{TF_{i}}\right)}^{2}\right)}^{2}}$$
Total muscle torque error	The root-mean square of the absolute difference between the estimated total muscle activation ($T_{MF}$) and the optimized total muscle activation ($T_{MI}$). $N_{s}$ is the number of samples.	$$e_{T}=\sqrt{\frac{1}{N_{s}}\sum_{i=1}^{N_{s}}{\left|T_{MI_{i}}-T_{MF_{i}}\right|}^{2}}$$
Processor usage percentage	The ratio of the time spent executing a program ($t_{program}$) to the total available processing time ($t_{total}$) expressed as a percentage. In multi-core processors, the summation of the percentage used by each processing core (*i*) is taken. $N_{p}$ is the number of processing cores.	$$P_{u}=\sum_{i=1}^{N_{p}}\left(\frac{t_{program_{i}}}{t_{total_{i}}}\cdot 100\%\right)$$
Program space	The summation of the number of bytes ($b_{i}$) per instruction (*k*) and bytes ($b_{d}$) per data value (*j*) of computer memory that a program requires to execute its behaviour. *I* and *D* are the total number of instructions and data values, respectively.	$$S_{p}=\sum_{k=1}^{I}b_{i_{k}}+\sum_{j=1}^{D}b_{d_{j}}$$
Data point optimization time	The total amount of time required to performance the optimization task ($T_{opt}$) divided by the number of data points ($N_{dp}$). This metric allows for comparison given different dataset lengths.	$$T_{dp}=\frac{T_{opt}}{N_{dp}}$$

**Table 4:** Description of elbow motions used in the experimental evaluation.

Motion (Motion ID)	Motion Description
Elbow flexion-extension (FE)	From full elbow extension, the subject flexes to 120${}^{{}^{\circ}}$, pauses and then extends until reaching full elbow extension.
Elbow extension-flexion (EF120)	Beginning at 120${}^{{}^{\circ}}$ flexion, the subject extends the elbow until reaching full extension, pauses and then flexes the elbow back to 120${}^{{}^{\circ}}$.
Elbow flexion-extension while holding a mass (FEM)	With a 1-kg mass held in the hand, the subject flexes from full extension to 120${}^{{}^{\circ}}$, pauses and returns to full extension.
Elbow flexion-extension with pauses (FEP)	With the elbow at full extension, the subject flexes until reaching 120${}^{{}^{\circ}}$, pauses and extends his/her elbow back to full extension. The subject also pauses once at some point during the flexion portion and once at some point during the extension portion of the motion. The pausing angles are chosen randomly by the subject.
Elbow flexion-extension with varying starting angles (FES)	The subject begins at a chosen flexion angle, proceeds to flex his/her elbow until reaching 120${}^{{}^{\circ}}$, pauses and then extends until reaching full extension. For each of the three repetitions, the subject started at 20${}^{{}^{\circ}}$, 60${}^{{}^{\circ}}$ and 100${}^{{}^{\circ}}$, respectively.

**Table 5:** Comparison of the total muscle activation error, total muscle torque error and data point optimization time metrics.

	Total Muscle Activation Error (×10^−3^)	Total Muscle Torque Error (Nm)	Data Point Optimization Time (μs)	Number of Datasets
**Model ID**	**Model Averages**			
Model 1	**0.706 ± 0.947** *	2.15 ± 1.42	**99.75 ± 17.69**	78
Model 2	**0.706 ± 0.947** *	2.15 ± 1.42	**156.09 ± 23.42**	78
Model 3	0.835 ± 1.086	2.10 ± 1.49	**255.56 ± 212.66**	78
Model 4	0.901 ± 1.151	2.17 ± 1.50	**766.40 ± 803.18**	78
Model 5	**0.946 ± 1.128** *	**2.19 ± 1.47** *	**301.88 ± 329.06** *	78
Model 6	0.702 ± 1.064	**1.67 ± 0.74** *	**266.61 ± 116.58** *	78
Model 7	**0.974 ± 1.179** *	**2.19 ± 1.50** *	**375.38 ± 356.94**	78
**Motion ID**	**Motion Averages**			
FE	0.684 ± 0.516	1.83 ± 0.83	392.17 ± 621.58	42
EF120	**0.718 ± 0.654** *	1.88 ± 0.87	326.37 ± 430.08	126
FEM	**1.533 ± 1.875** *	**2.84 ± 2.21** *	295.84 ± 342.42	126
FEP	**0.622 ± 0.450** *	**2.02 ± 1.03** *	350.04 ± 511.41	126
FES	**0.489 ± 0.358** *	**1.71 ± 0.87** *	281.55 ± 288.17	126
**Number of Parameters**	**Number of Parameters Averages**			
1	**0.706 ± 0.944**	2.15 ± 1.42	**127.92 ± 35.02**	156
2	**0.871 ± 1.121**	2.06 ± 1.38	**393.17 ± 471.87**	390
**Total**	0.824 ± 1.075	2.09 ± 1.39	317.38 ± 416.73	546

Bolded cells indicate statistically-significant results. Bolded cells that include an * indicate results where not all pairwise tests were statistically significant.
